# Supplementary material for: Identification of photoperiod-induced specific miRNAs in the adrenal glands of Sunite sheep (Ovis aries)
Source: Front Vet Sci. 2022 Jul 22;9:888207. doi: 10.3389/fvets.2022.888207 (PMC9354845; doi:10.3389/fvets.2022.888207)
Supplement: Supplementary file 1 [file Data_Sheet_1.ZIP › Supplementary file/miRDeep2 file.zip!/miRDeep2 file/Know_Structure/LP42A2/oar-mir-148a.pdf]

miRBase precursor : oar-mir-148a
Total read count : 1849111
oar-miR-148a read count : 1845228
remaining reads : 3883

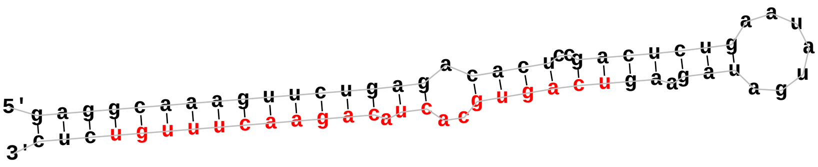

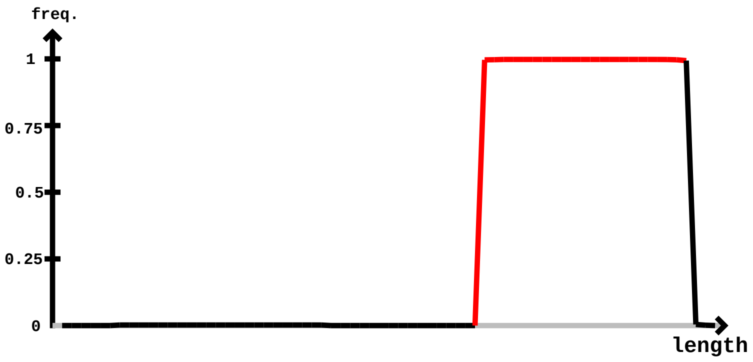

oar-miR-148a

5'- gaggcaaaguucugagacacuccgacucugaauaugauagaagucagugcacuacagaacuuugucuc -3'	exp		
((((((((((((((((.((((..(((((((.......))).))))))))..)).))))))))))))))	reads	mm	sample
....caaaguucugagacacuccga...	2	0	seq
....caaaguucugagacacuccgac..	2	0	seq
....caaaguucugagacacuccgacu...	69	0	seq
.....aaaguucugagacacuccg..	3	0	seq
.....aaaguucugagacacuccga...	71	0	seq
.....aaaguucugagacacuccgac..	30	0	seq
.....aaaguucugagacacuccgacu...	3684	0	seq
.....aaaguucugagacacuccgacuc..	4	0	seq
.....aaaguucugagacacuccgacucu.......................................	3	0	seq
.....aaaguucugagacacuccgacucug......................................	1	0	seq
......aaguucugagacacuccgacu...	12	0	seq
......aaguucugagacacuccgacucu.......................................	1	0	seq
...................cuccgacucugaauaugauagaag.........................	1	0	seq
....gucagugcacuacagaacuuu.....	1	0	seq
....gucagugcacuacagaacuuug....	1	0	seq
....gucagugcacuacagaacuuugu...	79	0	seq
....ucagugcacuacagaac........	8	0	seq
....ucagugAacuacagaacu.......	1	1	seq
....ucaCugcacuacagaacu.......	4	1	seq
....ucagugcacuacagaacu.......	173	0	seq
....ucaguAcacuacagaacu.......	1	1	seq
....ucagugcacuacagaacuu......	371	0	seq
....ucagugcacuacagaacuuu.....	1868	0	seq
....uUagugcacuacagaacuuu.....	6	1	seq
....ucagugcacuacagaacuuug....	5681	0	seq
....uUagugcacuacagaacuuug....	7	1	seq
....uUagugcacuacagaacuuugu...	2582	1	seq
....ucagugcacuacagaacuuugu...	1823857	0	seq
....ucagugcacuacagaacuuuguc..	5023	0	seq
....uUagugcacuacagaacuuuguc..	4	1	seq
....ucagugcacuacagaacuuugucu.	2648	0	seq
....ucagugcacuacagaacuuugucuc	10	0	seq
....cagugcacuacagaacuu......	1	0	seq
....cagugcacuacagaacuuu.....	1	0	seq
....cagugcacuacagaacuuug....	2	0	seq

oar-miR-148a

```
gaggcaaaguucugagacacuccgacucugaauaugauagaagucagugcacuacagaacuuugucuc
............................................Uagugcacuacagaacuuugu...    10      1      seq
............................................cagugcacuacagaacuuugu...    1105    0      seq
............................................cagugcacuacagaacuuuguc..    6       0      seq
............................................cagugcacuacagaacuuugucu.    4       0      seq
.............................................agugcacuacagaacuuu.....    1       0      seq
.............................................agugcacuacagaacuuug....    7       0      seq
.............................................agugcacuacagaacuuugu...    1736    0      seq
.............................................agugcacuacagaacuuuguc..    6       0      seq
.............................................agugcacuacagaacuuugucu.    1       0      seq
..............................................gugcacuacagaacuuugu...    10      0      seq
................................................Acacuacagaacuuugu...    1       1      seq
................................................gcaUuacagaacuuugu...    1       1      seq
................................................gcacuacagaacuuugu...    8       0      seq
................................................gcacuacagaacuuugA...    1       1      seq
................................................gcacuacagaacuuugG...    1       1      seq
................................................gcacuacagaacuuuguc..    1       0      seq
```
